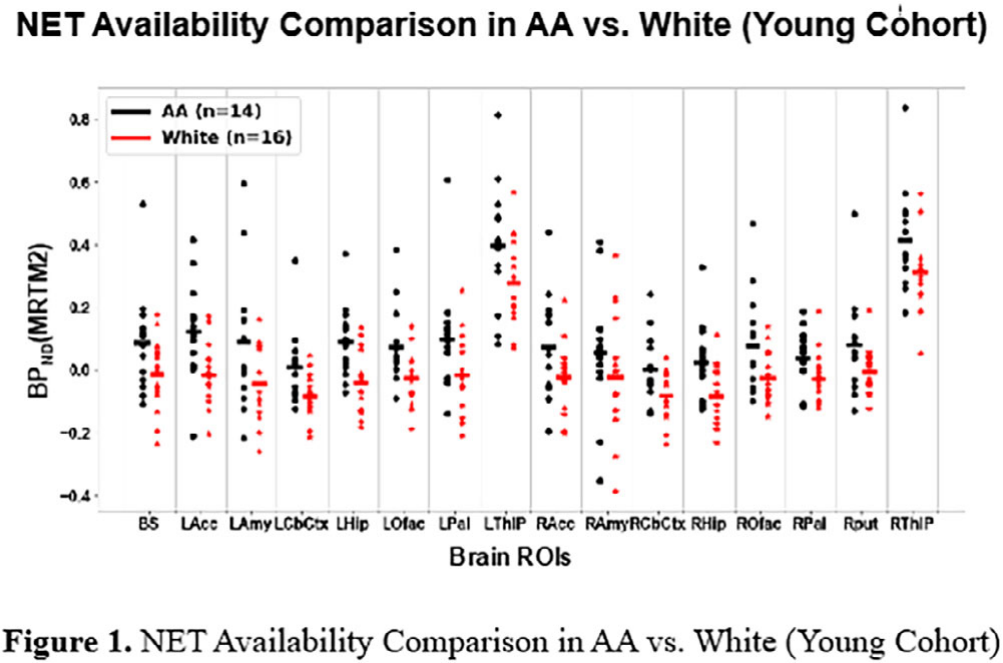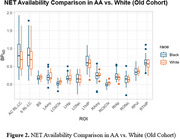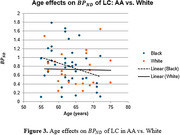# PET/MR imaging of LC‐NE function for interrogation of health disparity in preclinical AD

**DOI:** 10.1002/alz70856_105090

**Published:** 2026-01-07

**Authors:** Anmol S Bhatia, Andrew Kelleher, Ujjval Chopra, Jennifer Bernal, Sachita Gupta, Matthew Arthur, Oliver Cesar, Artem Mikheev, Jingyun Chen, Henry Rusinek, Yu‐Shin Ding, Ding's Lab

**Affiliations:** ^1^ NYU Grossman School of Medicine, New York, NY, USA; ^2^ Columbia University Irving Medical Center, New York, NY, USA

## Abstract

**Background:**

Locus Coeruleus (LC) is the earliest location for tauopathy and its decline is highly correlated with AD symptomatology. The LC‐NE system produces functional adaptive changes in response to chronic stress. [^11^C]MRB‐PET allows in vivo quantification of NE transporter (NET) in humans. Our previous data (ages 25‐55) demonstrated that African Americans (AA) had greater NET availability but with faster decline rate compared to whites (W), suggesting chronic lifetime discrimination/stress may contribute to health disparity. The goal of this study is to use a larger sample size and older cohort (55‐75) to further compare age and race effects on NET.

**Methods:**

Subjects underwent a 90‐min dynamic [^11^C]MRB‐PET via simultaneous PET/MRI. The segmentation of ROIs and LC delineation using TSE‐neuromelanin was established via registration of PET, MRI, and Freesurfer via Firevoxel (https://wp.nyu.edu/Firevoxel), Binding potential (BP_ND_, index of specific binding) using MRTM2 (t*=20 min, k2’=0.022 min‐1) and annual percent change (APC = 100 × (em – 1), m is slope) were calculated.

**Results:**

Participants (38 AA, age: 62.97±4.82; 23 W, age 64.63±5.71) were included in our old cohort. In young cohort, avg BP_ND_ values of ROIs (16) from AA (age 34±7) were consistently higher than those from W (Figure 1), while in old cohort, BP_ND_ in LC, thalamus, hippocampus, amygdala from AA remains higher, and the group difference was reduced (Figure 2).

In young cohort adults, all ROIs showed larger negative APC in BP_ND_ for AA, particularly in AA‐M, e.g., ‐3%/yr in LTH & RAmyg and ‐1%/yr in BS & ROfac; while ‐0.1 & ‐0.12%/yr in BS & ROfac for W. However, in old cohort, only LC and olfactory bulb showed larger negative APC in AA. Figure 3 illustrates that AA had a steeper decline in LC (slope ‐0.026) vs W (‐0.002).

**Conclusions:**

NET availability is higher in young AA and declines faster in old AA. These two competing factors result in a reduced group difference seen in old cohort. We demonstrate that LC‐NE function is sensitive to both age and stress effects and confirm that [^11^C]MRB‐PET is a promising strategy for early detection of underlying mechanisms for the health disparity in preclinical AD.